# CRMP2 and CRMP4 Are Differentially Required for Axon Guidance and Growth in Zebrafish Retinal Neurons

**DOI:** 10.1155/2018/8791304

**Published:** 2018-06-21

**Authors:** Zhi-Zhi Liu, Jian Zhu, Chang-Ling Wang, Xin Wang, Ying-Ying Han, Ling-Yan Liu, Hong A. Xu

**Affiliations:** ^1^Institute of Life Science, Nanchang University, Nanchang, China; ^2^School of Life Sciences, Nanchang University, Nanchang, China; ^3^Jiangxi Provincial Collaborative Innovation Center for Cardiovascular, Digestive, and Neuropsychiatric Diseases, Nanchang, China

## Abstract

Axons are directed to their correct targets by guidance cues during neurodevelopment. Many axon guidance cues have been discovered; however, much less known is about how the growth cones transduce the extracellular guidance cues to intracellular responses. Collapsin response mediator proteins (CRMPs) are a family of intracellular proteins that have been found to mediate growth cone behavior *in vitro*; however, their roles *in vivo* in axon development are much less explored. In zebrafish embryos, we find that CRMP2 and CRMP4 are expressed in the retinal ganglion cell layer when retinal axons are crossing the midline. Knocking down CRMP2 causes reduced elongation and premature termination of the retinal axons, while knocking down CRMP4 results in ipsilateral misprojections of retinal axons that would normally project to the contralateral brain. Furthermore, CRMP4 synchronizes with neuropilin 1 in retinal axon guidance, suggesting that CRMP4 might mediate the semaphorin/neuropilin signaling pathway. These results demonstrate that CRMP2 and CRMP4 function differentially in axon development *in vivo*.

## 1. Introduction

The correct formation of neural circuits is critical for establishing a functional nervous system. Axons grow out of neurons and usually travel a long distance to reach their correct targets. The axons must navigate accurately through the brain by following a precise path and the course is regulated by guidance cues [1, 2]. Dozens of axon guidance cues, including semaphorins, have been identified in the past decades. However, the intracellular molecular response mechanisms underlying how the growth cone of axons interpret environmental guidance cues are relatively less understood.

The collapsin response mediator protein (CRMP) is an intracellular protein discovered in a screen for components of the semaphorin 3A (originally named collapsin [3]) signaling pathway that mediates the collapse response of the growth cone [4]. Concurrently, a number of CRMPs were identified independently and referred to as turned on after division (TOAD-64) [5], dihydropyrimidinase-related protein (DRP or DPYSL) [6], unc-33-like protein (Ulip) [7], or TUC (TOAD64/Ulip/CRMP) [8]. In vertebrates, five members of CRMPs, CRMP1–5, have been identified [9, 10]. CRMPs are highly expressed in the nervous system at an early developmental stage and the expression dramatically drops in adults [9, 11]. CRMPs have been shown to be critical for many neurodevelopmental processes, such as neurogenesis, neuronal migration, and dendrite development [12]. Additionally, CRMPs might also be involved in axon regeneration, neurodegenerative diseases, and neuropsychiatric diseases [9, 12, 13]. Vertebrate CRMPs are homologous with *C. elegans unc-33*, the mutation of which results in severe defects of axon growth and guidance [14, 15]. Many studies have demonstrated that CRMPs are involved in growth cone collapse and axon growth *in vitro* [8, 10, 13, 16]. However, their roles in axon development *in vivo*, particularly in the central nervous system, still remain unclear. For example, in CMRP4 knockout mice, the apical dendrites of the CA1 pyramidal neurons are found to bifurcate precociously [17]. However, no anatomical or macroscopic changes in gross brain anatomy is observed in CMRP4 knockout mice [17, 18] although a selective decrease of axon extension and reduced growth cone area are observed in the cultured hippocampus neurons of CMRP4 knockout mice [18].

In the present study, the visual system was used to study the roles of CRMPs in axon development in the central nervous system. Both CRMP2 and CRMP4 were highly expressed in the retinal ganglion cell layer when retinal axons were crossing the chiasm and approaching the tectum in zebrafish. The two CRMPs functioned differentially in axon guidance and growth. CRMP2 was critical for axon elongation while CRMP4 was important for axon guidance. We also showed that CRMP4 synergized with neuropilin 1 in retinal axon guidance, suggesting that it might mediate the semaphorin/neuropilin signaling pathway.

## 2. Materials and Methods

### 2.1. Zebrafish Maintenance

Adult zebrafish (*Danio rerio*) were maintained in our facility on a 14–10 light–dark cycle in circulating water at 26–28°C. Embryos of either sex were raised at 28.5°C in E3 embryo medium (5 mM NaCl, 0.17 mM KCl, 0.33 mM CaCl_2_, and 0.33 mM MgSO_4_). Embryos to be collected for imaging were treated with 0.003% phenylthiourea (Sigma-Aldrich, St. Louis, USA) in E3 to prevent pigmentation. Embryos were staged by time after fertilization. All handling procedures were approved by the local ethical review committee at Nanchang University.

### 2.2. Whole-Mount In Situ Hybridization

The cDNAs used as templates to make probes were prepared by RNA extraction and reverse transcription PCR (RT-PCR). The primers for RT-PCR were designed according to the Esembl genomic sequences [19]. In situ hybridization was performed as described previously [20] with minor modifications. Briefly, DIG-labeled riboprobes were incubated with the embryos to detect the expression pattern of CRMP2 and CRMP4. Anti-DIG-alkaline phosphatase Fab fragments (Roche, Mannheim, Germany) and NBT/BCIP (Sangon Biotech, Shanghai, China) were used to detect and amplify the signals.

### 2.3. Morpholino Microinjections

Antisense morpholinos (MOs) were obtained from Gene Tools LLC (Philomath, OR, USA). MO sequences are as follows: CRMP2, 5′-CTT CTT GCC CTG ATA GCC AGA **CAT** C-3′ [21] (underlined nucleotides corresponding to the first initiation codon); CRMP4, 5′-TCT TTT TGC CTT GGT AAG A**CA T**GG T-3′ [22]; and Nrp1a, 5′-GAA TCC TGG AGT TCG GAG TGC GGA A-3′ [20, 23]. The knockdown effects and specificity of CRMP2 and CRMP4 MOs have been validated by rescuing the resulting phenotypes with the corresponding mRNAs [21, 22]. Nrp1a MO has also been validated in an *in vitro* transcription and translation system [23] and *in vivo* in axon guidance [20]. The sequence of the standard control MO from Gene Tools is 5′-CCT CTT ACCTCA GTT ACA ATT TAT A-3′. Approximately 1–1.5 nl of MOs at different dosages were injected into embryo yolk at the 1-2 cell stage.

### 2.4. Retinal Axon Labeling

Retina axons were labeled by dye injection as previously described [24]. Briefly, zebrafish larvae were fixed at 4 dpf (days post fertilization) in 4% PFA. Retinal axons were labeled by injecting lipophilic dye DiI into one eye and DiD into the other eye. The dye-labeled retinotectal axons were scanned on an Olympus FV1000 confocal microscope and all images were presented as maximum projections of the z series. The number of the eyes were counted with normal or abnormal retinal axon guidance or growth. Fisher's exact test was used to compare the effects of the combination of the morpholinos with that of the sum of the single half doses of morpholinos.

## 3. Results

### 3.1. CRMP2 and CRMP4 Were Expressed in a Retinal Ganglion Cell Layer When Retinal Axons Were Crossing the Midline

The visual system is a classical modeling system to study axon guidance of the central nervous system since retinal axons travel a long distance through the brain [25]. Retinal axons exit the eye and extend ventrally to cross the midline at the optic chiasm. They continue to extend dorsally and posteriorly to the tectum. In contrast to binocular animals, all retinal axons cross the midline and project to the contralateral brain in zebrafish.

**Figure 1:**
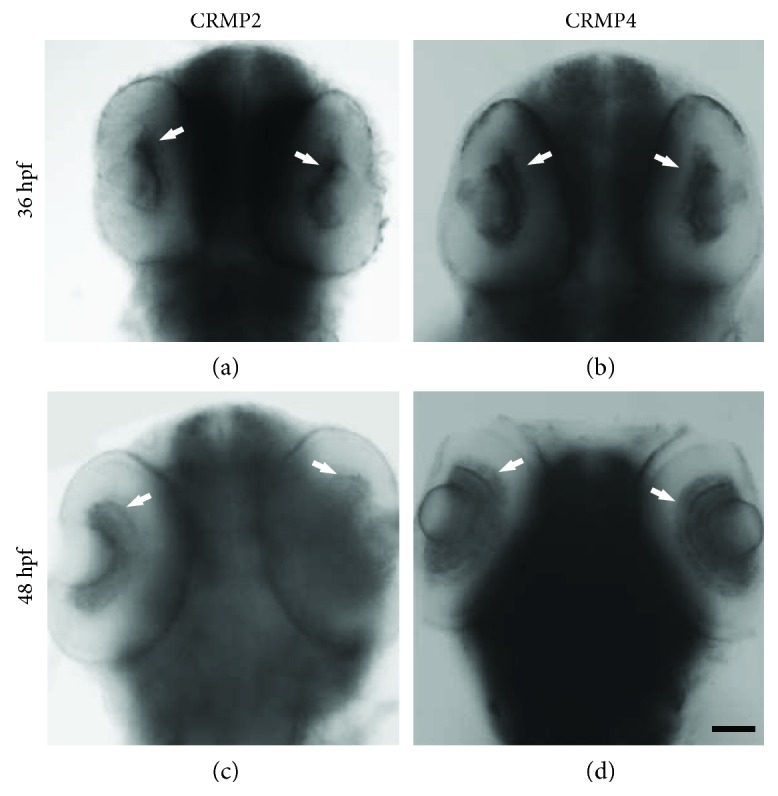
CRMP2 and CRMP4 are expressed in the retinal ganglion cell layer when retinal axons are exiting the eye and crossing the midline. Whole mount in situ hybridization was performed in zebrafish embryos using RNA probes for CRMP2. (a, b) CRMP2 and CRMP4 transcripts are detected in the retina (white arrows) at 36 hpf. (c, d) CRMP2 and CRMP4 transcripts are detected in the retina of 48 hpf embryos (white arrows). Scale bar: 50 *μ*m.

In order to investigate the role of CRMPs in axon growth and guidance *in vivo*, we performed in situ hybridization with antisense probes for CRMPs in zebrafish embryos. Our preliminary results revealed that CRMP1–4 were all expressed in the retina at 36 hours post fertilization (hpf). We focused on CRMP2 and CRMP4 since CRMP2 is the most studied member of the CRMP family and both CRMP2 and CRMP4 have been shown to be critical for axon regeneration. CRMP2 and CRMP4 were highly expressed in the retinal ganglion cell layer surrounding the lens at 36 hpf (Figures 1(a) and 1(b)), when the first retinal axons are exiting the eye and crossing the midline. Both of the CRMPs were still highly and specifically expressed in the retina at 48 hpf, when retinal axons are approaching and starting to arborise the tectum (Figures 1(c) and 1(d)). Besides being expressed in the retina, CRMP2 and CRMP4 were also highly expressed in specific brain regions and a subpopulation of neurons in the spinal cord, consistent with previous reports [11, 26]. We confirmed the expression patterns of CRMP2 and CRMP4 by using antisense probes targeting nonoverlapping regions of the mRNA transcripts. The spatiotemporal expression patterns of CRMP2 and CRMP4 in the retinal ganglion cell layer suggested that they might be important for axon growth and guidance *in vivo*.

### 3.2. Knocking Down CRMP2 Resulted in Growth Defects of Retinal Axons

**Figure 2:**
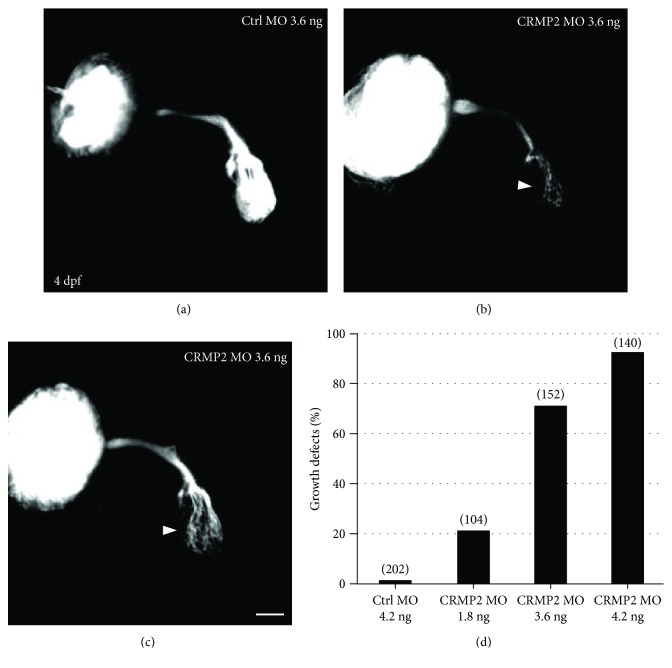
Knocking down CRMP2 induces growth defects of retinal axons. Morpholino was injected into the zygotes at the 1-2 cell stage. Embryos were allowed to grow until 4 days (4 days postfertilization, 4 dpf) and fixed with PFA. Lipophilic fluorescent dye DiI or DiD was injected into an eye of the larvae to label retinal axons. (a) An example showing that, in control MO-treated zebrafish larvae, retinal axons exit the eye, cross the midline, and grow into and arborize the whole tectum at 4 dpf. (b, c) Representative images of retinal axons of CRMP2 MO-treated embryos. Much less retinal axons grow into and arborized the tectum (white arrowheads) compared with that in control MO-treated embryos. (d) The growth defects of retinal axons induced by CRMP2 MOs are dose-dependent. The *y*-axis represents the percentage of eyes with growth defects of retinal axons. The doses of MOs are labeled under each column. The numbers in parentheses above each column indicate the amount of eyes. Scale bar: 50 *μ*m.

CRMP2 has been shown to be critical for axon specification and growth *in vitro* [8, 16]; however, its roles *in vivo* still remain unclear. We injected morpholino (MO), an antisense oligonucleotide sequence specifically targeting the transcribed mRNA, into zebrafish zygotes to block the translation of CRMP2 [21]. Fluorescence lipophilic dyes were injected into the eyes of fixed embryos to label retinal axons. Normally, the first retinal axons reach and start to arborize the optic tectum at 2 dpf and a preliminary arborization of the tectum is formed at 3 dpf (data not shown). At 4 dpf, many retinal axons have arborized the whole tectum (Figure 2(a)). However, in CRMP2 MO-treated embryos (morphants), much less retinal axons arborized the tectum in morphants compared to wildtype embryos. Some retinal axons might terminate prematurely and fail to reach the tectum even at 4 dpf, although many retinal axons grew out of the eye and formed the optic tract (Figures 2(b) and 2(c)). The growth defects of retinal axons in CRMP2 morphants were dose-dependent, with much more severe defects at higher doses of morpholino (Figure 2(d)). The reduced arborization of the tectum in morphants suggested that CRMP2 might be critical for axon elongation, consistent with the reports *in vitro* [8, 16]. CRMP2 has long been presumed to be involved in axon guidance since its discovery. However, we only found rare axon guidance errors, such as ipsilateral misprojections (2%) and dorsal misprojections (2.6%) in CRMP2 morphants (data not shown). In most morphants, despite growth defects, the residue retinal axons still crossed the midline, followed normal optic pathways, and projected correctly into the tectum (Figures 2(b) and 2(c)). These results revealed that CRMP2 was critical for axon elongation *in vivo*.

### 3.3. Knocking Down CRMP4 Caused Retinal Axons to Misproject Ipsilaterally

**Figure 3:**
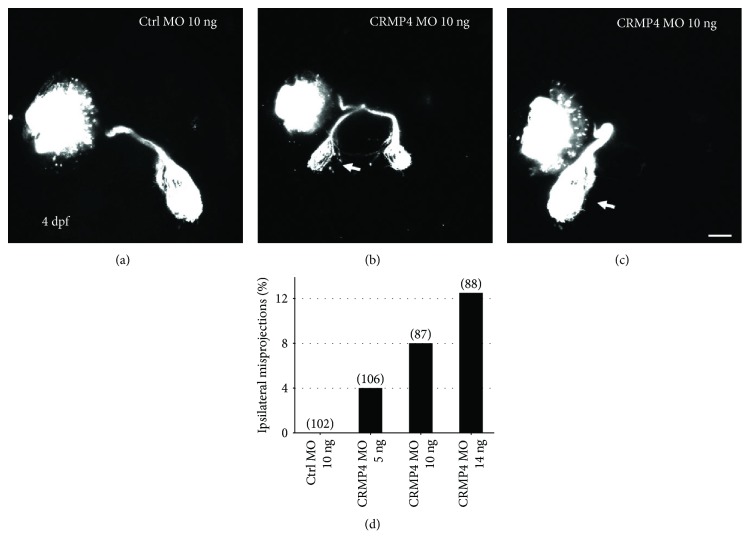
Knocking down CRMP4 causes ipsilateral misprojections of retinal axons. Morpholino was injected into 1-2 cell stage embryos and retinal axons were labeled with DiI or DiD at 4 dpf. (a) A representative image demonstrating that, in control MO-treated zebrafish larvae, all retinal axons cross the midline and project into the opposite side of the tectum. (b, c) Representative images of retinal axon guidance errors in CRMP4 MO-treated larvae. A part or all of the retinal axons fail to cross the midline and misproject into the ipsilateral tectum (arrows). Note that although the axons misproject ipsilaterally, they still follow the normal optic tract and arborize into the tectum. (d) The ipsilateral misprojections of retinal axons caused by CRMP4 MOs are dose dependent. The doses of MOs are labeled under each column. The numbers in parentheses above each column indicate the amount of eyes. Scale bar: 50 *μ*m.

Similar to CRMP2, CRMP4 was also highly expressed in the retinal ganglion cell layer. However, different from CRMP2 morphants, only mild growth defects of retinal axons were found in CRMP4 morphants (data not shown). Unexpectedly, knocking down CRMP4 resulted in axon guidance defects. In control MO-treated larvae, nearly all retinal axons crossed the midline and projected into the contralateral tectum (Figure 3(a)). However, in CRMP4 morphants, some retinal axons failed to cross the midline and misprojected into the ipsilateral tectum (Figures 3(b) and 3(c)). The axon guidance errors caused by CRMP4 MO were dose-dependent (Figure 3(d)). The ipsilaterally misprojected retinal axons still follow the correct orbit of the normal optic tract, suggesting that CRMP4 mainly mediates axon crossing at the midline. These results demonstrated that CRMP2 and CRMP4 functioned differentially in axon guidance and growth during the development of the visual system.

### 3.4. CRMP4 Synergized with Neuropilin 1 in Retinal Axon Guidance

**Figure 4:**
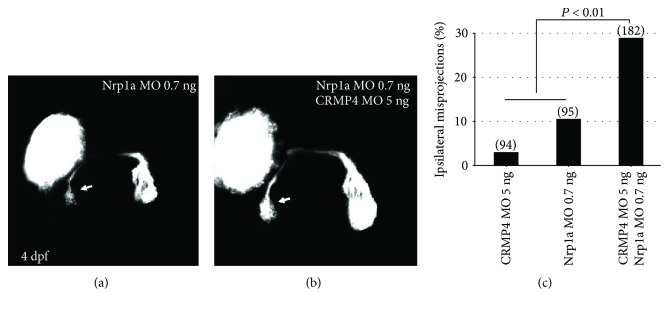
CRMP4 and Nrp1a synergize in retinal axon guidance. A low dose of either CRMP4 or Nrp1a induce a small percentage of ipsilateral misprojections. A half dose of either CRMP4 MOs or Nrp1a MOs was injected singly or in combination. (a, b) Representative images of knocking down effects by a half dose of Nrp1a MOs or Nrp1a and CRMP4 in combination. Some retinal axons fail to cross the midline and misproject ipsilaterally (arrows). (c) The combination of the two morpholinos induce a significantly higher percentage of ipsilateral misprojections than simply adding up the misprojections caused by the two half doses (Fisher's exact test, *P* < 0.01). The doses of MOs are labeled under each column. The numbers in each column indicate the amount of eyes.

The ipsilateral misprojection phenotypes caused by CRMP4 knockdown was reminiscent of the phenotypes induced by knocking down Sema3D, Sema3E, and their coreceptor neuropilin 1 (Nrp1) [20]. This suggested that CRMP4 might mediate the signaling transduction of Sema3/Nrp1 in axon guidance. In order to test this possibility, we knocked down Nrp1 and CRMP4 with half doses of MOs. At half doses of either the CRMP4 or Nrp1a MO, only a low percentage of ipsilateral misprojections was observed (Figure 4(a)), much less than that caused by full doses of either corresponding MO. The combination of half doses of CRMP4 and Nrp1a induced a much higher percentage of ipsilateral misprojections than the sum by adding up that induced by single half doses of them (Figures 4(b) and 4(c)). This indicated that CRMP4 and Nrp1a synergize with each other in retinal axon guidance, suggesting that they might function in a common signaling pathway. This is consistent with the *in vitro* studies that CRMPs function downstream of semaphorin/neuropilin [27–29].

### 3.5. CRMP2 and CRMP4 Synergize with Each Other in Axon Growth but Not Axon Guidance

**Figure 5:**
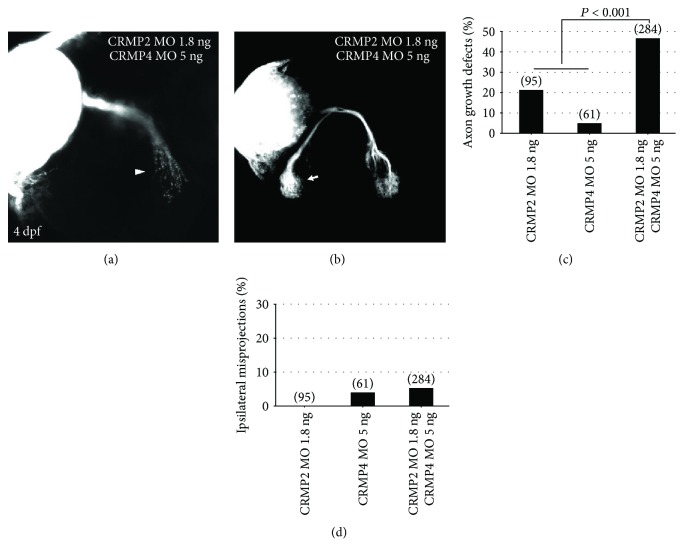
CRMP2 synergized with CRMP4 in axon growth but not axon guidance. A half dose of either CRMP2 or CRMP4 MOs was injected singly or in combination. (a, b) Representative examples of knocking down effects of CRMP2 and CRMP4 MOs in combination. (c) The combination of the two MOs causes a significantly higher percentage of axon growth defects than the sum of the defects caused by adding up the single half doses (Fisher's exact test, *P* < 0.001). (d) No obvious synergy between the two CRMPs in retinal axon guidance. The doses of MOs are labeled under each column. The numbers in parentheses above each column indicate the amount of eyes.

The above results suggested that CRMP2 and CRMP4 might play differential roles in axon growth and guidance. It seemed that CRMP2 might mainly function in axon growth while CRMP4 participates in axon guidance. Since both of the two CRMPs were expressed in the retinal ganglion cell layer, we asked whether they cooperate with each other in axon development. We found that combining half doses of CRMP2 and CRMP4 MOs resulted in more axon growth defects than the sum of the defects caused by the two single half doses of morpholinos (Figure 5). The synergy between CRMP2 and CRMP4 suggested that they participate in axon growth in a common signaling pathway. However, we did not observe any synergy between the two CRMPs in retinal axon guidance (Figure 5(d)). The synergy between the two MOs in axon growth but not axon guidance suggested that the observed phenotypes could not be due to any toxic effect but instead due to their specific effects. These data further demonstrated that CRMP2 and CRMP4 played differential roles in axon development. CRMP2 might mainly participate in axon elongation while CRMP4 mainly function in axon guidance. Additionally, CRMP4 might coordinate with CRMP2 in axon elongation.

## 4. Discussion

It has long been proposed that CRMPs might play critical roles in axon development since their discovery; however, little *in vivo* evidence has been presented. In this study, we report that CRMP2 and CRMP4 are critical for retinal axon guidance and growth in zebrafish embryos. Interestingly, the two CRMPs function differentially in axon development: CRMP2 mainly participates in axon outgrowth and elongation while CRMP4 seems to play dual roles in both axon guidance and growth.

Vertebrate CRMPs share a homology with the UNC-33 protein in the nematode *C. elegans*. Mutations in the *unc-33* gene cause severe axon extension defects and guidance errors [14, 15]. Our current findings that knocking down CRMPs results in defects of axon growth and guidance suggest that the roles of vertebrate CRMPs in axon development are well conserved evolutionally between invertebrates and vertebrates. It has been demonstrated that CRMP2 regulates axon initiation and elongation *in vitro* [16, 30]. Those findings are consistent with our results that both the number and length of retinal axons are reduced in the tectum of the CRMP2 morphants. The bundle of retinal axons in the optic nerve and optic tract seems to be thinner than that in wild type embryos, suggesting possible initiation failure of axons; however, further study is required to confirm the possibility. Some of the retinal axons terminate prematurely and fail to reach the tectum (Figures 2(b) and 2(c)), indicating the failure of axons to elongate further into the tectum in the absence of CRMP2. Besides the visual system, CRMP2 might also be broadly required for axon growth in other neural systems [31]. Furthermore, in the adult mouse hippocampal dentate gyrus, there are axonal growth and targeting defects in newborn granule neurons with CRMP2 knockdown [32]. This indicates that axon growth during embryonic development and adult newborn regeneration might share some common mechanisms related to CRMP2. CRMP4, similar to CRMP2, has been revealed to be involved in axon growth *in vitro* and *in vivo* [18, 22]. All these findings are consistent with our results, although CRMP4 seems to play only a minor role in axon growth as compared to CRMP2.

Since their discovery, CRMPs have been proposed to be involved in axon guidance by mediating the activity of semaphorins and other guidance cues. In the present study, we demonstrate that CRMP4 is critical for the proper direction of retinal axons in the chiasm. Knocking down CRMP4 causes retinal axons to misproject to the ipsilateral side of the brain. The ipsilateral misprojections of retinal axons are reminiscent of those caused by knocking down Sema3s or neuropilin 1 [20, 33]. Furthermore, the synergetic effect between CRMP4 and neuropilin 1 (Figure 4) indicates that CRMP4 might function downstream of the semaphorin/neuropilin signaling pathway in guiding axons [20]. However, the intracellular domain of neuropilin is very short (about 20 amino acids long). So it is reasonable to speculate that CRMP4, as an intracellular protein, should interact with a transmembrane receptor for semaphorin, such as plexin. Further studies are guaranteed to search for such a receptor and investigate its interaction with neuropilin and CRMP4.

It is intriguing that CRMP2 and CRMP4 cooperate in axon growth while playing differential roles in axon guidance. Continuous remodeling of the neuronal cytoskeleton is critical for axon extension and turning. Microtubules and actin filaments are central to the coordinated control of the cytoskeleton in the growth cone of the axons. It has been demonstrated that CRMP2 and CRMP4 similarly interact with tubulin while differentially involved in actin dynamics [18, 34, 35]. The cooperated and differential roles of CRMP2 and CRMP4 in axon development could partially be due to their complex interactions with tubulin and actin. The differential roles of CRMP2 and CRMP4 in axon development could also be associated with other factors, such as differential expression of CRMPs upon stimulation [36], differential response to various axon guidance cues [37], specific phosphorylation by different kinases [36, 38], and subcellular distribution [39].

Alternative splicing of CRMPs (CRMP1–4) has been shown to result in two isoforms that differ in the first exons and consequently different N-termini: the short isoforms (CRMP1–4S (*S*mall or *s*hort)) and the isoforms that are longer by ~100 amino acids (CRMP1–4L (*L*arge or *l*ong)) [40, 41]. It has been shown that the alternatively spliced isoforms of CRMP2 are differentially involved in axon guidance and growth [42]. Our PCR results suggest that the zebrafish CRMP2 and CRMP4 also have alternatively spliced isoforms (data not shown). However, we fail to distinguish the expression pattern of the short and long isoforms. The morpholinos used in this report are translational blockers targeting the short isoforms [22], CRMP2S and CRMP4S. Further studies are required to investigate the roles of the long isoforms of CRMPs in axon growth and guidance *in vivo*.

## 5. Conclusion

The study has revealed the intracellular mechanisms of how the CRMPs transduce the extracellular guidance cues into behavioral responses of the growth cone *in vivo*. CRMP2 and CRMP4 are expressed in the retinal ganglion cell layer when retinal axons are growing from the eye to the brain. CRMP2 and CRMP4 differentially participate in axon development *in vivo*. CRMP2 mainly mediates axon elongation while CRMP4 mediates axon guidance. We also demonstrate that CRMP4 phenocopies neuropilin 1a in retinal axon guidance and they synergize with each other, suggesting that they might mediate the semaphorin/neuropilin signaling pathway *in vivo*. Our findings will help to understand the underlying molecular mechanisms of axon development and regeneration and CRMP-related diseases such as neurodegeneration and neuropsychiatric disorders.
